# Immunohistochemistry as a method to study elastic fibers of human vocal fold

**DOI:** 10.1016/S1808-8694(15)31204-0

**Published:** 2015-10-20

**Authors:** Hugo Valter Lisboa Ramos, Manuel de Jesus Simões, Paulo Augusto de Lima Pontes, Luciano Rodrigues Neves, Luiz Henrique Fonceca Barbosa, Noemi Grigoletto De Biase, Celina T.S. Oshima

**Affiliations:** 1 Otorhinolaryngologist (Post-graduate studies under course).; 2 Ph.D. (Professor, UNIFESP-EPM).; 3 Ph.D. in Medicine (Doctorate thesis), Escola Paulista de Medicina, 1970. Ph.D. in Medicine, Course of Post-graduation in Medicine, 1981. Full Professor, Discipline of Otorhinolaryngology, Department of Ophthalmology/Otorhinolaryngology, Escola Paulista de Medicina, 1989. Faculty Professor, Department of Otorhinolaryngology and Human Communication Disorders, Escola Paulista de Medicina, 1991.; 4 Post-graduation under course, UNIFESP-EPM.; 5 Otorhinolaryngologist (Post-graduation under course).; 6 Ph.D.; 7 Ph.D. (Biologist).

**Keywords:** larynx, vocal fold, elastin, immunohistochemistry

## Abstract

**Aim**: Verify the use of immunohistochemistry as a method to measure all forms of elastic fibers at human vocal folds. **Study design:** transversal cohort. **Material and method**: We collected vocal folds following these criterion: age between 25 and 40, Caucasian men, dead by gun shot, within 12 hours of death, without instrumentation of the larynx or suspicion of neck injury and without mucosal lesions noted by microscopy. Ten vocal folds were collected and one, of a man aged 28 years, was selected to study. The vocal fold was transversely cut in 9 regions and in each segment three slides were made. These slides were stained by Verhoeff and Weighert´s resorcin-fuchsin and used for immunohistochemistry. The elastic compound was measured by colorimetric software analysis. **Results**: In Verhoeff and Weighert´s resorcin-fuchsin, the intermediate and deep layer showed values higher than those of the superficial layer. The amount of tropoelastin identified by the antibody at the superficial layer was close to those of intermediate and deep layer. **Conclusions**: Immunohistochemistry is a method that can identify and measure all forms of elastic fibers at human vocal fold.

## INTRODUCTION

The physiology of sound production by the vocal folds maintain a close relation with the characteristics of vocal fold lamina propria (LP). Hirano^1^ established a correlation between trilaminar structure of LP and the vibration process and highlighted the importance of the superficial layer, constituted of loose tissue with few collagen and elastic fibers, of intermediate layer (CI), comprising elastic fibers.

The identification of elastic fibers up to the year 1997 was carried out by subjective analyses, based on the observation of the researcher. This year, Hammond et al. ^2^ presented a study using a system of colorimetric quantification of elastic tissue stained by Verhoeff technique and they compared the results with electron microscopy (ME). By means of this system, that allows statistical analysis of the results, the authors demonstrated that the elastic fibers are preferably concentrated in the lamina propria CI.

ME has also been used by authors such as Ishii et al.^3^ and Sato & Hirano^4^ and currently it is considered as the method that best identifies the different fibrous proteins of vocal fold. It presents great specificity in the identification of elauninic and oxitalanic elastic fibers that is based on their shape. However, this method does not allow quantification of structures.

To the extent of our study, there is no other study that used immunohistochemistry to identify the elastic fibers of human vocal fold. Similarly to ultrastructural method, the antibody allows that elastic fibers be identified in all its shapes. However, it allows the quantification of fibers in a very specific way, without interference of other structures that may also be stained.

The purpose of the present study was to check the applicability of the immunohistochemical method in the quantification of different forms of elastic fibers in human vocal folds.

## MATERIAL AND METHOD

This experimental study was development in the Department of Otorhinolaryngology and Head Neck Surgery, Pathology and Morphology, UNIFESP - EPM. The study was approved by the Research Ethics Committee, Federal University of Sao Paulo/ Hospital Sao Paulo.

We followed the inclusion criteria below: age between 25 and 40 years, male gender, Caucasian, death caused by firearm, less than 12 hours after death, absence of tracheal intubation and neck trauma that in the microscopic analysis did not present any vocal fold mucosa affection. According to these criteria, ten vocal folds were randomly obtained and one was sectioned, which belonged to a 28-year-old subject.

The vocal fold was placed in formaldehyde solution at 10% buffer for tissue fixation and later it was processed by the usual technique of paraffinization. After being impregnated with paraffin, the vocal fold was transversally sectioned in nine regions. The fragments were extended to the anterior extremity of the vocal fold - named fragment 1 - up to the posterior extremity, together with the vocal fold of the arytenoid cartilage - fragment 9.

Out of each fragment of the vocal fold we removed 3 sections - 4 micrometers thick each, to make the slides for the histological and immunohistochemical study. Two sections of each fragment were used to make the staining with Verhoeff and resorcin-fuchsin Weighert and the third section was used to make the immunohistochemical study. We used polyclonal antibody against human tropoelastin, which is a soluble subunit responsible for the formation of the amorphous portion of elastin (Midwood^5^), obtained from serum of immunized rabbits, manufactured by the laboratory EPC (PR398, Polyclonal Antiserum to Human Tropoelastin). To perform the immunohistochemical study, we used antibody at the dilution of 1:800, obtained after the pilot study.

Slides were analyzed with a histomorphometric study comparing the intensity of colors in the superficial, intermediate and deep layers of lamina propria. To locate the layers of lamina propria and the choice of the histological field to be analyzed we used some histological references. The superficial layer was defined as tissue which was homogenously located in the adjacent area of the epithelium and that had very clear deep margins. The intermediate layer was defined as the homogenous layer that was placed in the adjacent area of the superficial layer, and the deep layer was defined as the region of lamina propria that maintained closed relation with the thyroarytenoid muscle, given that the limits between the intermediate and deep layers did not show clear borders by the histological studies and immunohistochemical techniques. The limits were initially defined in an enlargement of 40 times.

The images were captured with a digital camera (Sony Hyper HAD) coupled to a light microscope (Laboval 4) and connected to a computer with a board Captvator, using 400x magnification. The image was processed by the software DIRACOM3 developed by the Laboratory of Information Technology Dedicated to Dental Sciences (LIDO), Department of Stomathology, Dental School, University of Sao Paulo and currently updated and commercialized under the name Imagelab 2000^®^ (Novelli^6^).

**Figure 1:**
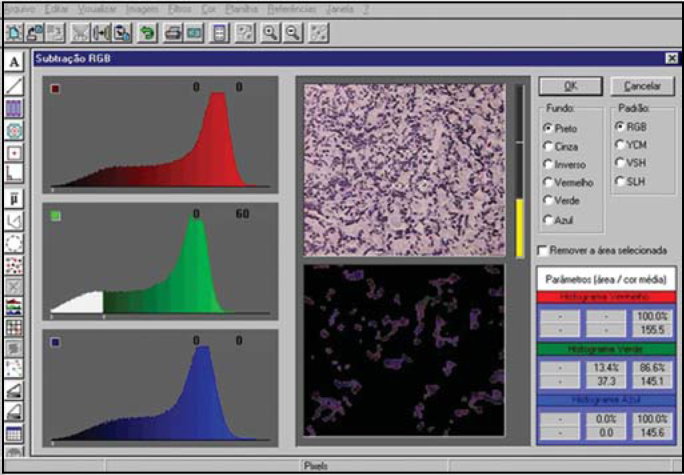
Image of software Imagelab 2000 informing the selected color (central quadrant to the left), original image (central upper quadrant), subtracted (central lower quadrant) and percentage of involved areas relative to the original image (right lower quadrant).

This software allows the identification, selection and subtraction of structures of images by means of spectrums R (*red*), G (*green*) and B (*blue*), using 126 tones of red, 126 of blue and 126 of green. The higher the number of red, blue and green tones and their combinations in studied tissue, the higher the likelihood of separating structures within their limits. The computer has a screening of all images, comparing the value of the color of each pixel with a table of preciously selected patterns. If the value corresponds to some value presented in the table, it is subtracted from the image so that they maintain only structures formed by pixels different from those that belong to the selected pattern. At the end, the computer informs the percentage of removed areas in relation to the area of the original image (Figure 1).

In this study, we used green to select pixels that corresponded to the elastic fibers in different staining. Verhoeff and resorcin-fuchsin Weighert staining were measured in black and immunohistochemistry was measured in brown.

The results were descriptively analyzed.

## RESULTS

Tables 1, 2 and 3 show the amount of elastic tissue present in the vocal fold LP that could be measured in different staining techniques. The values found were distributed according to the region of the vocal fold and with the LP studied layer. The values corresponded to the percentage of the area of colors selected by the software Imagelab 2000^®^ in relation to the area of the original image.

**Table 1:** Mean percentage of the area occupied by black color on the slides stained with Verhoeff on superficial, intermediate and deep layers of vocal fold on anterior, medium and posterior thirds.

	Superficial	Intermediary	Deeplayer
**Anterior**	6,9	23,5	29,7
**Medium**	4,5	21,3	17,1
**Posterior**	10,4	23,3	25,0

**Table 2:** Mean percentage of the area occupied by black color on the slides stained with resorcin-fuchsin of Weighert on superficial, intermediate and deep layers of vocal fold on anterior, medium and posterior thirds.

	Superficial	Intermediary	Deeplayer
**Anterior**	1,2	12,8	10,4
**Medium**	0,7	13,7	10,8
**Posterior**	0,5	7,8	5,5

**Table 3:** Mean percentage of the area occupied by brown color on the slides stained with polyclonal antibody against human tropoelastin on superficial, intermediate and deep layers of vocal fold on anterior, medium and posterior thirds.

	Superficial	Intermediary	Deeplayer
**Anterior**	8,8	12,2	10,1
**Medium**	7,3	12,0	12,3
**Posterior**	9,3	10,7	11,2

**Figure 2:**
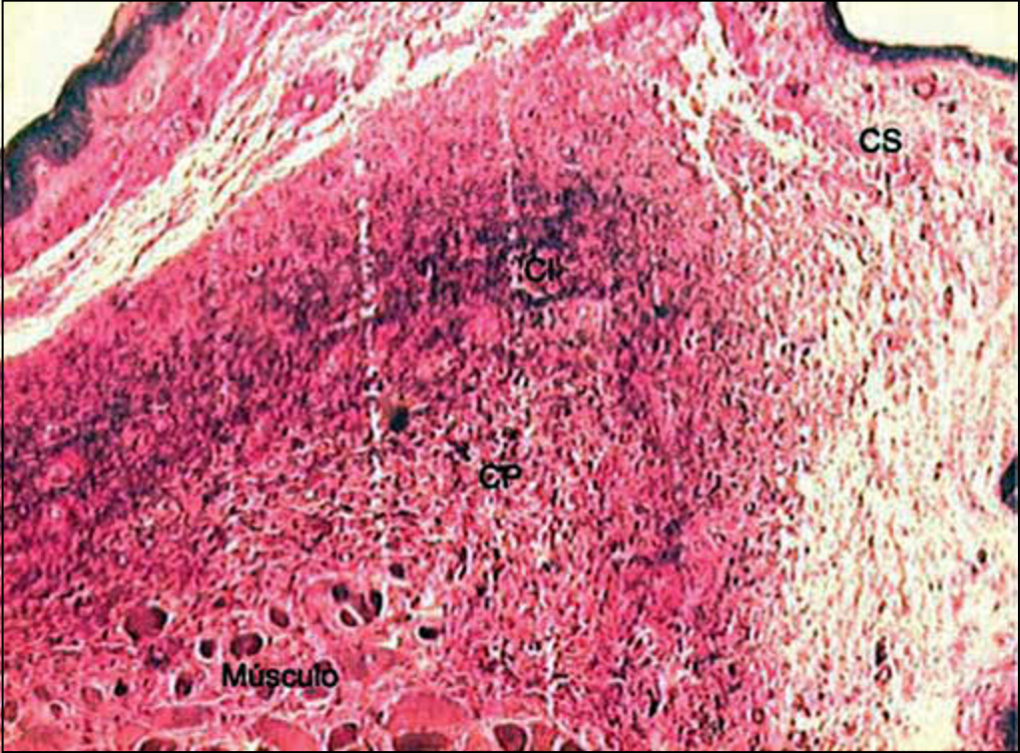
Distribution of elastic fibers in the lamina propria layers stained by Verhoeff. (Fragment - 3, magnification 40 X). CS - superficial layer, CI - intermediate layer, CP - deep layer.

**Figure 3:**
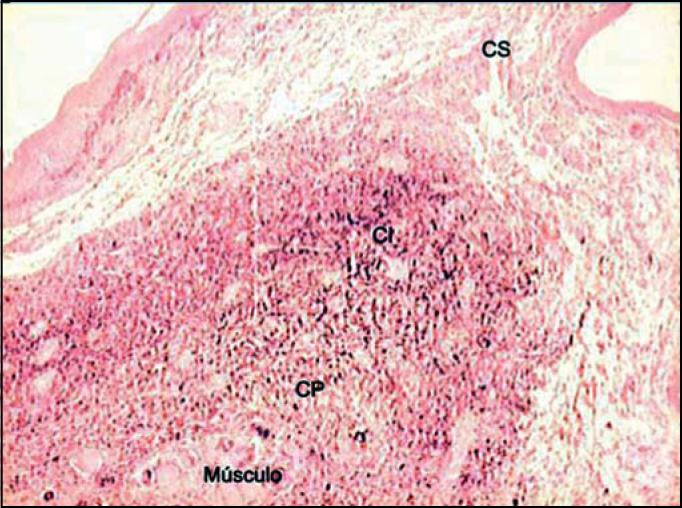
Distribution of elastic fibers on the lamina propria layers stained with resorcin-fuchsin of Weighert. (Fragment - 3, magnification 40 X). CS - superficial layer, CI - layer intermediate, CP - layer deep.

**Figure 4:**
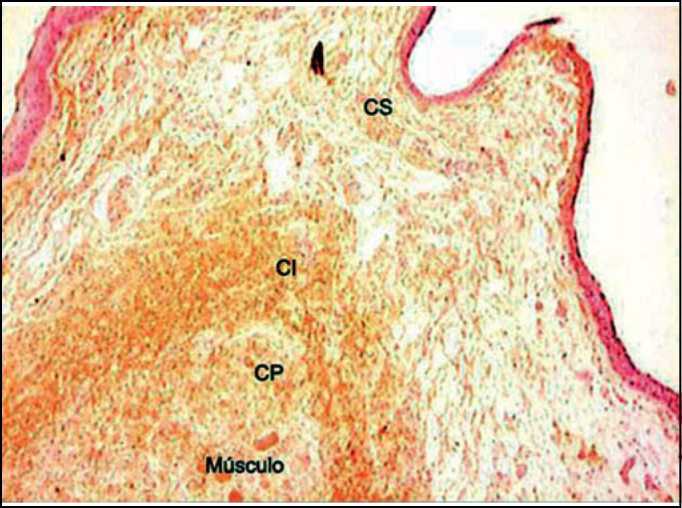
Distribution of tropoelastin on lamina propria layers. (Fragment - 4, magnification 40 X). CS - superficial layer, CI - intermediate layer, CP - deep layer.

Figures 2, 3, and 4 showed distribution of elastic fibers in superficial, intermediate and deep layers, in staining Verhoeff, resorcin-fuchsin of Weighert and immunohistochemical.

Analyzing the values obtained in the Verhoeff and resorcin-fuchsin of Weighert staining we could see that the intermediate and deep layers of the vocal fold present very higher values than the superficial layer in the different regions of the vocal fold. However, we could notice that the amount of tropoelastin identified by the antibodies in the superficial layer of LP was closer to the values of intermediate and deep layers.

**Figure 5:**
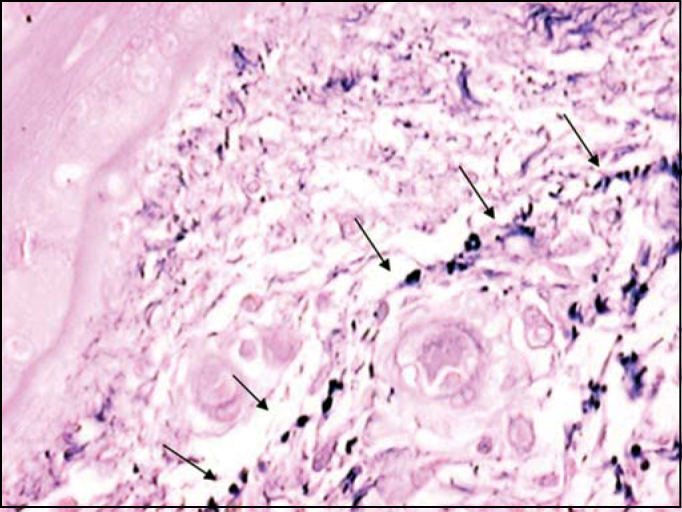
Plexus of elastic fibers (arrows) parallel to basal membrane stained with resorcin-fuchsin of Weighert. (Fragment - 4, magnification 400 X).

**Figure 6:**
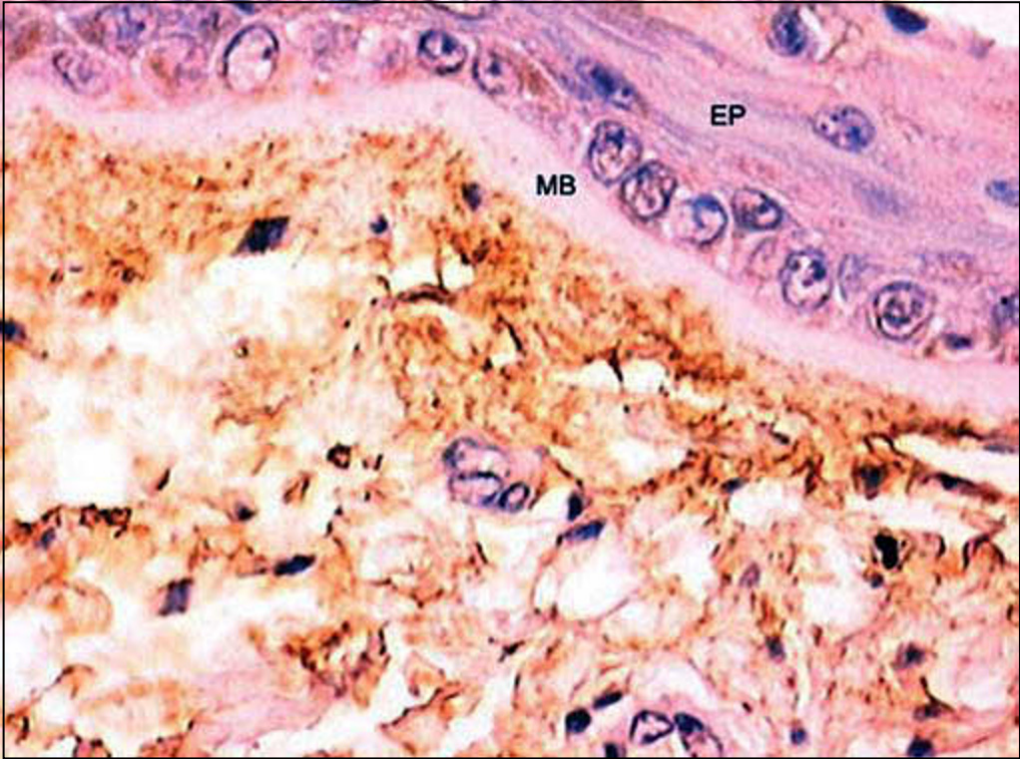
Identification of tropoelastin adjacent to basal membrane. (Fragment - 4, magnification 400 X). EP - epithelium, MB - basal membrane.

Figure 5 shows, by resorcin-fuchsin of Weighert staining, the presence of the network of elastic fibers that occurs right below the basal membrane. It is interesting to notice that this plexus and basal membrane of the epithelium show a space in which there is almost no stained elastic tissue, and there are only two small and rare black stained points. However, upon observing Figure 6 we detect that the antibody could identify tropoelastin in this region located adjacent to the basal membrane.

## DISCUSSION

Since the pioneer work by Hirano ^1^, LP has attracted the attention of laryngologists in the whole world. Upon proposing the theory of phonation body and cover, Hirano launched the basis over which the whole physiology of vibration of the vocal fold has been supported. Through electron microscopy, he demonstrated that the medium layer of LP is formed primarily by elastic fibers and that the number is decreased towards the deep layer.

Trying to study the role of elastic fibers in the formation and propagation of mucosa wave of the vocal fold, Hammond et al. ^2^ used a system of image analysis to measure the amount of elastic tissue stained with Verhoeff in 40 human larynges and compared the findings with the study by electron microscopy carried out in 6 human larynges. Confirming the results of Hirano, the authors observed that elastic fibers are specially concentrated on the intermediate layer of the vocal fold. They also described that there are innumerous small fibers on the LP superficial layer, which, according to the authors, would correspond to immature forms of elastic fibers: elaunin and oxitalan. It is believed that because they do not suffer distension and are less resistant to mechanical stress, oxitalanic fibers are abundant on the superficial layer.

Montes^7^, upon studying the elastic fibers in different human tissues, demonstrated that oxitalan is located in areas submitted to mechanical stress: periodontum, dermal-epidermal junction, endoneurum, ciliary body of the eye, etc. In human skin, oxitalanic fibers are displaced in a perpendicular shape to the dermal-epidermal junction, together to the basal membrane and connected to a plexus of elauninic fibers, which are also bound to deeper mature elastic fibers. The three types of elastic fibers belong to a continuum in which oxitalan, elaunin and mature elastic fibers contained collections of microfibrils, with growing intensity of amorphous substance. Even though this sequence corresponds to successive steps of formation of elastic fiber, the author believes that the presence of oxitalanic and elauninic fibers in tissues in which, even if fully mature, do not contain completely formed elastic fibers suggests that these fibers have a specific structural role.

Differently from those authors, which supported electron microscopy for the morphological study of vocal fold elastic fibers, our study used three types of staining: Verhoeff and resorcin-fuchsin of Weighert, already used before, and a third type not applied for this purpose, which is immunohistochemistry.

Montes^7^ described that immature elastic fibers are more difficult to be stained by usual methods and demonstrated that Verhoeff is only capable of staining fibers that contain amorphous substance and, for this reason, it manages to demonstrate only complete elastic fibers. This author also showed that the only chemical staining method that managed to identify elauninic and oxitalanic fibers was resorcin-fuchsin of Weighert, and he said that oxitalanic fibers suffered strong previous oxidation.

Traditionally, oxitalan has been described as a bundle of microfibrils of about 10-12nm in diameter that does not contain amorphous substance. However, Schwartz & Fleischmajer^8^ demonstrated, with immunofluorescence of specific antibodies for elastin and microfibrils, that oxitalan is associated with a small amount of the amorphous substance of human skin.

The amorphous substance, called elastin protein, is formed by tropoelastin molecules that are secreted by the cell to the extracellular space and make covalent binds with other subunits. In our study, we demonstrated that the antibody is capable of identifying tropoelastin, and as a consequence, amorphous substance in all layers and in the whole lamina propria of the human vocal fold. Oxitalanic fibers are believed to be located there and immunohistochemistry showed the presence of elastic tissue (Figure 6). Ishii et al. ^3^ had already demonstrated, with the use of electron microscopy, that oxitalan is located right below the squamous epithelium of the human vocal fold, similarly to what we find on the skin.

Differently from EM, which identified the structures by revealing the geometric shape of different components of the studied tissue, immunohistochemistry makes it based on the capacity the antibody has to bind to the structure of which it is formed. The structures might not have the same molecular characteristics with which the antibody reacts, not being stained and, thus, not identified. This major specificity allows that the tissue studied by immunohistochemistry be submitted to colorimetric measurements not performed by EM.

We have also observed, using resorcin-fuchsin staining of Weighert, the presence of a plexus of elastic fibers that runs parallel and right below the basal membrane (Figure 5). We think that this network of elastic fibers corresponds to elauninic fibers responsible for the connection of deep mature elastic fibers to subepidermal oxitalanic fibers.

We know that freedom of movement of the vocal fold cover is closely related with amount of vibration tissue and freedom of movement in all directions of the LP and that the structure of the extracellular matrix and the appropriate distribution of the elastic fibers may be decisive in this process.

To the extent we managed to study, this is the only analysis that uses immunohistochemistry in the identification of elastic fibers of human vocal folds and our results show that this technique may be applied to this end comprising all evolution forms of the elastic fibers. New studies with more human vocal folds should be performed so that we develop deep knowledge about the role of elastic fibers in biomechanical systems involved in vocal production. We think that the measurement of elastic fibers using antibody may provide this information.

## CONCLUSION

The obtained results allowed us to conclude that immunohistochemistry is a technique that identifies all forms of elastic fibers in human vocal folds and that it also enables the performance of objective measurements.
